# Glucagon-like Peptide-1 receptor agonists for the prevention and treatment of Parkinson’s disease

**DOI:** 10.1017/S109285292510031X

**Published:** 2025-06-09

**Authors:** Serene Lee, Liyang Yin, Naomi Xiao, Taeho Greg Rhee, Heidi K.Y. Lo, Sabrina Wong, Susan Fox, Kayla Teopiz, Bess Yin-Hung Lam, Yang Jing Zheng, Gia Han Le, Rodrigo B. Mansur, Joshua D. Rosenblat, Roger S. McIntyre

**Affiliations:** 1 Department of Research, Brain and Cognition Discovery Foundation, Toronto, ON, Canada; 2 Department of Health Sciences, Queen’s University, Kingston, ON, Canada; 3 Department of Psychiatry, Yale School of Medicine, New Haven, CT, USA; 4 Department of Public Health Sciences, University of Connecticut School of Medicine, Farmington, CT, USA; 5 Department of Psychiatry, School of Clinical Medicine, LKS Faculty of Medicine, The University of Hong Kong, Hong Kong, P. R. China; 6 Department of Psychiatry, University of Toronto, Toronto, ON, Canada; 7 Mood Disorder Psychopharmacology Unit, University Health Network, Toronto, ON, Canada; 8 Department of Counseling and Psychology, Hong Kong Shue Yan University, Hong Kong, P. R. China; 9 Department of Pharmacology and Toxicology, University of Toronto, Toronto, ON, Canada; 10 Institute of Medical Science, University of Toronto, Toronto, ON, Canada

**Keywords:** Parkinson’s disease, glucagon-like peptide-1 receptor agonist, diabetes, α-synuclein, exenatide, liraglutide

## Abstract

Parkinson’s disease (PD) is a severe neurodegenerative disorder characterized by prominent motor and non-motor (e.g., cognitive) abnormalities. Notwithstanding Food and Drug Administration (FDA)-approved treatments (e.g., L-dopa), most persons with PD do not adequately benefit from the FDA-approved treatments and treatment emergent adverse events are often reasons for discontinuation. To date, no current therapy for PD is disease modifying or curative. Glucagon-like peptide-1 receptor agonists (GLP-1RAs) are central nervous system (CNS) penetrant and have shown to be neuroprotective against oxidative stress, neuroinflammation, and insulin resistance, as well as promoting neuroplasticity. Preclinical evidence suggests that GLP-1RAs also attenuate the accumulation of α-synuclein. The cellular and molecular effects of GLP-1RAs provide a basis to hypothesize putative therapeutic benefit in individuals with PD. Extant preclinical and clinical trial evidence in PD provide preliminary evidence of clinically meaningful benefit in the cardinal features of PD. Herein, we synthesize extant preclinical and early-phase clinical evidence, suggesting that GLP-1RAs may be beneficial as a treatment and/or illness progression modification therapeutic in PD.

## Introduction

Parkinson’s disease (PD) is a chronic neurodegenerative disorder characterized by motor and non-motor abnormalities (e.g., cognitive impairments).^1^^,^^2^ Although the etiology of PD is not fully known, multiple risk factors have been identified.^3^ For example, type 2 diabetes mellitus (T2DM) is identified as a risk factor for PD insofar as approximately 10–30% of persons with PD have T2DM as a comorbid diagnosis.^4^^–^^7^ In addition, some molecular and cellular alterations, although non-specific, do in some cases overlap in PD and T2DM.^8^ For example, incretin deficits are observed in both conditions, and several studies have observed prophylactic effects against PD after the administration of incretin mimetics.^9^

The essential pathological features of PD are the loss of spontaneously firing tyrosine-hydroxylase (TH)-positive dopaminergic neurons in the substantia nigra pars compacta (SNpc) and the accumulation of α-synuclein aggregates (a molecular hallmark of PD) into Lewy bodies.^10^^–^^15^ PD is also associated with microglial activation, which triggers oxidative stress as evidenced by the presence of oxidative markers (e.g., reactive oxygen species).^13^

Although Food and Drug Administration (FDA)-approved treatments exist for PD, notably dopamine replacement therapies, no intervention is disease-modifying or curative.^16^ In addition, most persons with PD do not achieve optimal acute and long-term efficacy, tolerability, or safety.^17^^,^^18^ The unmet clinical needs in PD provide the impetus for alternative, mechanistically informed therapeutics that are effective, safe, and acceptable.^18^

Glucagon-like peptide-1 (GLP-1) receptors are expressed in the central nervous system (CNS) and exhibit neuroprotective and neuroplasticity-enhancing effects.^19^^,^^20^ The expression of GLP-1 receptors in the SNpc and striatum suggests that their pharmacologic modulation may affect the functioning and survival of neuronal populations in the SNpc and other dopaminergic target sites within the brain (e.g., prefrontal cortex).^19^^–^^21^ GLP-1 receptors are also observed in multiple cell types (e.g., microglia), which supports evidence for the anti-inflammatory properties of GLP-1RAs in PD, increasing cell viability.^22^^,^^23^ Moreover, preclinical and preliminary clinical data indicate that GLP-1 receptor agonists (GLP-1 RAs) attenuate nigral dopaminergic neuronal loss by inhibiting apoptosis.^21^

Glucagon-like peptide-1 receptor agonists are incretin mimetics that are FDA-approved for treating T2DM, obesity, and cardiovascular disease.^24^^,^^25^ In addition, GLP-1RAs reduce markers of neuroinflammation and oxidative stress, suggesting that these agents may protect or sustain cellular integrity and survival in brain regions implicated in PD (e.g., SNpc).^13^^,^^15^^,^^19^^–^^23^^,^^26^^,^^27^ In keeping with this view, a testable hypothesis is that GLP-1RAs may benefit clinical features and/or prevent the onset and progression of PD by targeting multiple aforementioned effector systems relevant to the viability and function of neurons. Herein, we synthesize the extant preclinical and clinical literature surrounding the conjecture that GLP-1RAs be administered for the prevention and treatment of PD and attenuate the interplay between T2DM and PD.

## Methods

### Search strategy

A comprehensive search was conducted across online databases, including Embase, PubMed, OVID Medline, and Google Scholar, from inception through July 24, 2024. Subsequent manual searches of the reference lists of the obtained articles were conducted. The following Boolean search string was used: (Parkinson’s disease) AND (GLP-1) OR (glucagon-like peptide-1) OR (exenatide) OR (liraglutide) OR (dulaglutide) OR (lixisenatide) OR (insulin degludec) OR (insulin glargine) OR (semaglutide) OR (tirzepatide). A second search string was also applied to ensure the search was fully comprehensive: (Parkinson’s disease) AND (GLP-1) AND ((treatment) OR (prevention)). Studies were limited to the language of publication (e.g., English).

Two independent reviewers (SL, LY) screened the articles obtained using the Covidence software.^28^ After removing duplicates, articles were screened by title, abstract, and full text against the eligibility criteria (Table 1). Any discrepancies in screening between reviewers were resolved by discussion.

**Table 1.** Eligibility Criteria

Inclusion criteria
A primary or post-hoc analysis Preclinical or clinical study Subjects must be administered with GLP–1RAs Subjects must have PD or MPTP (1-methyl–4-phenyl–1,2,3,6-tetrahydropyridine) or α-synuclein-induced Parkinsonian syndrome Clinical studies must be administered with FDA-approved GLP–1RAs Comorbidities (e.g., obesity, type 2 diabetes mellitus)
Exclusion criteria
Secondary data (e.g., meta-analysis, systematic review, narrative review) Full text unavailable online 3. Publication language not in English

### Data extraction

Extracted data were established a priori using a piloted data extraction table. Data extraction was conducted by one reviewer (SL). The extracted data included: (1) author(s) and publication year, (2) study design and participants, (3) intervention, (4) duration, (6) intervention outcome(s), and (7) significance of the study outcome(s) (Table 2).

**Table 2.** Descriptive Characteristics of Included Preclinical and Clinical Studies

Author(s)	Subjects	Subject criteria	Intervention	Duration	Intervention outcome	Significance
Preclinical studies						
Liu et al.^14^	Control (*n* = 12) MPTP/vehicle (*n* = 12) Ex–4 (Exendin–4) (*n* = 12) MPTP/Ex–4 (*n* = 12) Liraglutide (*n* = 12) MPTP/liraglutide (*n* = 12) Lixisenatide (*n* = 12) MPTP/ lixisenatide (*n* = 12)	8-week old male mice weighing 25–30 g injected with MPTP	Ex–4 (10 nmol/kg) or liraglutide (25 nmol/kg) or lixisenatide (10 nmol/kg) injected once daily	14 days	Ex–4 showed no protective effects at the dose chosen but liraglutide and lixisenatide prevented MPTP-induced motor impairment	Liraglutide and lixisenatide are superior to Ex–4
Zhang et al.^29^	56 mice randomly allocated to four groups (*n* = 14)	Adult male mice weighing 23 ± 2 g injected with MPTP	(Val8)GLP–1-glu-PAL (25 nmol/kg i.p.) once-daily	8 days	(Val8)GLP–1-glu-PAL reversed MPTP-induced motor impairment compared to the control. ANOVA found a significant difference in all groups (p < .0001)	Demonstrated neuroprotective effects of the new GLP–1 analogue: biomarkers and apoptosis were improved
Zhang et al.^12^	Control (*n* = 12) Liraglutide (*n* = 12) Semaglutide (*n* = 12) MPTP (*n* = 12) MPTP + liraglutide (*n* = 12) MPTP + semaglutide (*n* = 12)	10-week-old mice weighing 20–25 g injected with MPTP	Liraglutide (25 nmol/kg ip.) or semaglutide (25 nmol/kg ip.) once daily	7 days	ANOVA test found an overall difference between the control and experimental groups (p < .0001), where the GLP–1RAs were able to normalize the MPTP-induced locomotor impairments	Semaglutide is more effective than liraglutide in normalizing behavior
Cao et al.^30^	Control (*n* = 13) MPTP (*n* = 13) MPTP +25 nmol/ kg liraglutide (*n* = 13) MPTP +50 nmol/kg liraglutide (*n* = 13) MPTP +100 nmol/kg liraglutide (*n* = 13)	Male mice weighing 18–22 g injected with MPTP	Liraglutide (25 50, or 100 nmol/kg intraperitoneally once daily	9 days	Motor activity was significantly improved in the liraglutide groups compared to the control (p = .004) in the swimming test	GLP–1 analogues can induce sustained therapeutic effects
Bu et al.^31^	Number of total subjects is not stated	Three-month-old Sprague–Dawley female rats weighing 250–280 g injected with MPTP	Ex–4 (1.25 μg/mL dissolved in normal saline, 5 μg/kg/day) twice daily	10 weeks	Ex–4 significantly reduced asymmetrical movements in behavioral assessments compared to the control (p = 0.0092).	Ex–4 attenuated TH-positive neuronal loss in the SNpc, alleviating denervation and synaptic dysfunction. Ex–4 attenuates PD by autophagy pathways
Liu et al.^32^	Total *n* = 59	Adult male mice weighing 22–26 g injected with MPTP	Ex–4 (10 μM) once daily	Duration is not stated	Ex–4 significantly increased the firing rate of 7/14 partial nigral dopaminergic neurons from 2.20 ± 0.31 Hz to 3.54 ± 0.41 Hz	Spontaneous firing of nigral dopaminergic neurons is associated with important roles like neuronal survival, but is decreased in PD. Ex–4 stimulated neuronal firing
Kim et al.^27^	Saline (*n* = 5) Ex–4 (*n* = 5) Saline + MPTP (*n* = 5) Ex–4 + MPTP (*n* = 5)	8-week-old male mice weighing 22–24 g injected with MPTP	Ex–4 (10 mg/kg) 30 minutes prior to each MPTP injection for a total of four injections	1 week	Ex–4 administration prevented MPTP-induced loss of TH-positive SNpc neurons with an increase in cell viability by 83% (p < .01)	Ex–4 protects nigrostriatal dopamine pathways from Parkinsonian toxicity of MPTP by inhibition of microglial activation and concomitant release of microglia-derived pro-inflammatory mediators
Elbassuoni and Ahmed^15^	Control (*n* = 15) PD (*n* = 15) Diabetic PD (*n* = 15) Diabetic PD + low dose treatment (*n* = 15) Diabetic PD + high dose treatment (*n* = 15)	Adult male albino Sprague–Dawley rats weighing 150–200 g injected with MPTP	Exenatide (1.0 or 5.0 μg/kg) subcutaneously once daily	28 days	Catalepsy test scores were 6.9 ± 0.8 for control, 29.5 ± 4.5 for PD, 49.9 ± 5.6 for diabetic PD, 30.8 ± 4.8 for diabetic PD + low dose treatment, and 16.2 ± 2.7 for diabetic PD + high dose treatment	Subjects administered exenatide showed significantly improved striatal dopamine levels and significant improvements in catalepsy test scores compared to the control (p < .05)
Yun et al.^13^	Number of total subjects is not stated	Ten-month-old mice injected with α-syn PFF	NLY01 (3 mg/kg) injected subcutaneously twice a week	5 months	NLY01 protects *in vivo* against DA neuronal loss and behavioral deficits by α-syn PFF	GLP–1RA inhibition of microglia is the primary site of action for protection
Clinical studies						
Athauda et al.^33^	Treatment (*n* = 32) Placebo (*n* = 30)	Subjects aged 25–75 years with moderate, idiopathic PD, dopaminergic treatment with wearing-off effects, and were at Hoehn and Yahr stage 2:5 or less	Exenatide (2 mg) subcutaneously once weekly	60 weeks	Off-medication MDS-UPDRS scores improved by 1·0 points in the exenatide group and worsened by 2·1 points in the placebo group	Exenatide had positive effects in motor scores in PD which were sustained beyond the period of exposure
Meissner et al.^34^	Control (*n* = 78) Lixisenatide (*n* = 78)	Subjects aged 40–75 years old and had received a diagnosis of PD according to U.K. Brain Bank Criteria within the past 3 years	Lixisenatide (10 μg) for 14 days, then lixisenatide (20 μg) subcutaneously daily for the remainder of the 12-month period	12 months	At 12 months, MDS-UPDRS part 3 scores improved for the lixisenatide group (−0.04 points) and worsened for the placebo group (3.04 points)	Lixisenatide has an effect on motor disability progression related to a neuroprotective mechanism but did not improve non-motor symptoms
Brauer et al.^35^	Control (*n* = 38 393) Exposed GLP–1 (*n* = 10 684) Exposed GTZ (*n* = 21 175) Exposed DPP4 inhibitors (*n* = 36 897)	Subjects 18 years or older that received at least two consecutive prescriptions for a glucose-lowering agent	Administration of any GLP–1RA	Data was collected from patient medical records from inception until March 2019	The rate of PD was 36–60% lower in users of GLP–1RAs compared to users of other oral antidiabetic drugs	PD was effectively prevented in diabetic patients after treatment with GLP–1RAs compared to the control
Tang et al.^36^	GLP–1RA (*n* = 30 091) DPP4 inhibitors (*n* = 58 983)	Older adults with T2DM	Administration of any GLP–1RA	Data was collected from patient medical records from 2016 until 2020	The crude incidence rate of PD was lower among GLP–1RA users than DPP4i users (2.85 versus 3.92 patients per 1000 person-years)	PD was effectively prevented in diabetic patients after treatment with GLP–1RAs compared to DPP4i

### Quality assessment

Study quality was assessed by two independent reviewers (SL, LY). Preclinical studies were evaluated by the Systematic Review Centre for Laboratory Animal Experimentation (SYRCLE) Risk of Bias tool, randomized controlled clinical studies were evaluated using the Cochrane Risk of Bias Tool for Randomized Studies (RoB2), and observational cohort studies were evaluated using the Quality Assessment Tool for Observational Cohort and Cross-Sectional Studies, adapted from the National Institute of Health (NIH) guidelines (Tables 3–5).^37^^–^^39^

**Table 3.** Risk of Bias in the Assessment of Preclinical Studies

Study	1	2	3	4	5	6	7	8	9	10	Risk of bias judgements
Liu et al.^14^	Y	Y	Y	Y	U	Y	Y	Y	Y	Y	Low
Zhang et al.^29^	Y	Y	Y	Y	Y	Y	Y	Y	Y	Y	Low
Zhang et al.^12^	Y	Y	Y	Y	N	Y	U	Y	Y	Y	Low
Cao et al.^30^	Y	Y	Y	Y	U	Y	U	Y	Y	Y	Low
Bu et al.^31^	Y	Y	Y	Y	Y	Y	Y	Y	Y	Y	Low
Liu et al.^32^	Y	Y	Y	Y	Y	Y	Y	Y	Y	Y	Low
Kim et al.^27^	Y	Y	Y	Y	U	U	Y	Y	Y	Y	Low
Elbassuoni and Ahmed^15^	Y	Y	Y	Y	U	U	Y	Y	Y	Y	Low
Yun et al.^13^	Y	Y	Y	Y	Y	U	Y	Y	Y	Y	Low

**Table 4.** Risk of Bias Assessment of Randomized Controlled Clinical Studies

Study	1	2	3	4	5	Risk of bias judgements
Athauda et al.^33^	N	N	N	N	N	Low
Meissner et al.^34^	N	N	N	N	N	Low

## Results

### Study results and selection

**Figure 1.**
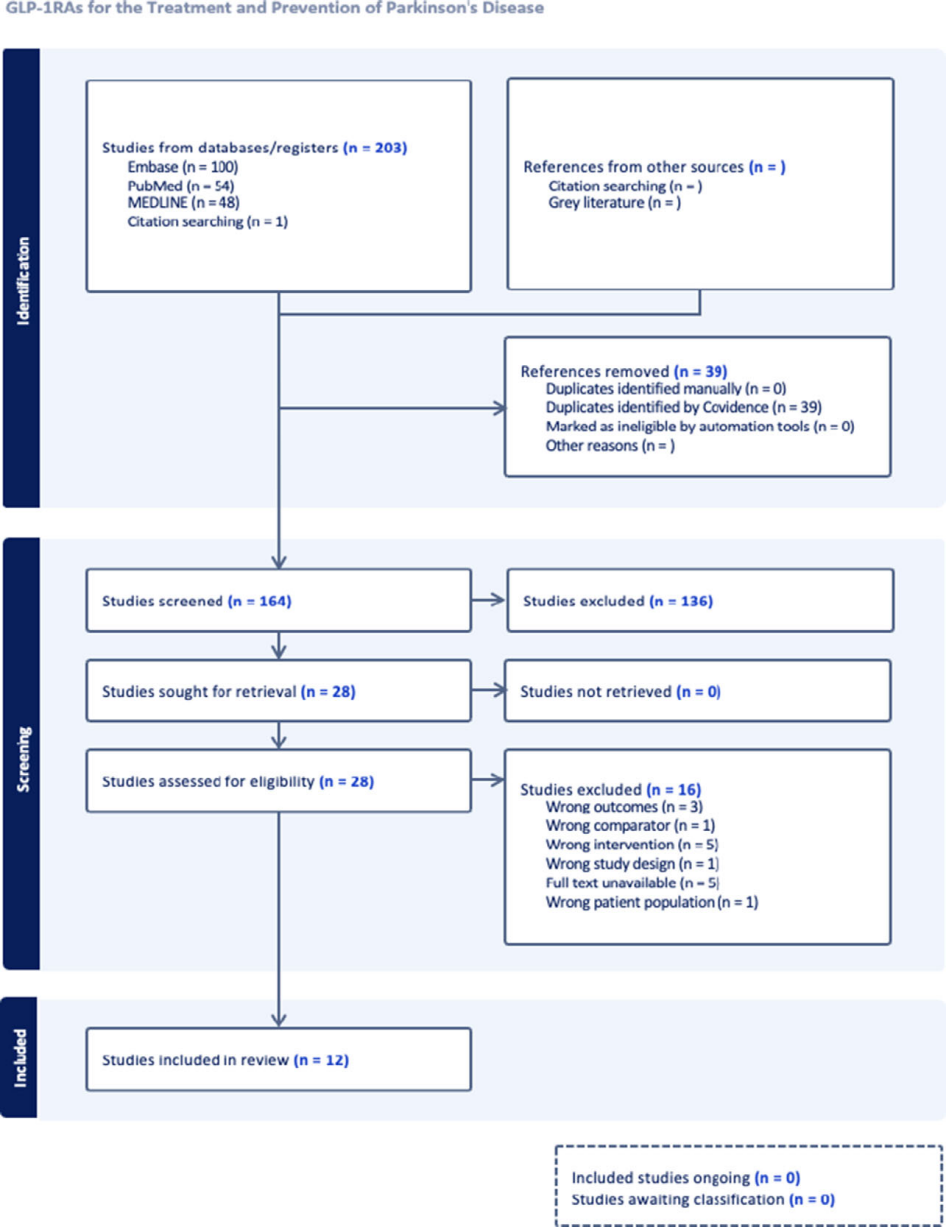
PRISMA flowchart of literature identification and selection.

The initial search generated a total of 203 publications from Embase, PubMed, OVID Medline, and citation searching as seen on PRISMA (Figure 1). After removing duplicates, 164 studies remained for title and abstract screening, of which 28 were deemed eligible for full-text screening. Full-text screening resulted in 12 eligible publications to be included in the systematic review. The majority of articles were excluded due to outcomes not aligned with the eligibility criteria of our analysis.

**Table 5.** Risk of Bias Assessment of Observational Cohort Clinical Studies

Study	1	2	3	4	5	6	7	8	9	10	11	12	13	14	Risk of bias judgements
Brauer et al.^35^	Y	Y	Y	Y	Y	Y	Y	N	Y	N	Y	NR	Y	Y	Good
Tang et al.^36^	Y	Y	Y	Y	Y	Y	Y	N	Y	N	Y	NR	Y	Y	Good

### Preclinical evidence of GLP-1RAs in PD

A total of nine preclinical studies were retrieved from the search. In all included preclinical studies, rodents were injected with 1-methyl-4-phenyl-1,2,3,6-tetrahydropyridine (MPTP) or α-synuclein to induce sporadic PD models. Liu et al.,^14^ Zhang et al.,^29^ and Zhang et al.^12^ reported that exendin-4 (Ex-4), liraglutide, lixisenatide, (Val8)GLP-1-glu-PAL, and semaglutide, respectively, exerted neuroprotective effects against MPTP-induced motor impairments such as bradykinesia, abnormal posture, and gait.^12^^,^^14^^,^^29^ Rotarod motor sensory performance, open-field distance, catalepsy, and swimming tests were conducted to evaluate locomotor and exploratory activity in mice.^12^^,^^14^^,^^29^ Liu et al.^14^ reported that liraglutide and lixisenatide reversed some of the identified MPTP-induced motor impairments, while Ex-4 did not.^11^ Additionally, Zhang et al.^12^ reported that semaglutide was more effective than liraglutide in reversing MPTP-induced motor impairment in mice (p < .001).^12^^,^^14^ Moreover, western blot analyses indicated that both (Val8)GLP-1-glu-PAL and semaglutide reversed neurodegeneration by decreasing levels of Bcl-2-associated X protein (BAX), an apoptosis-promoting molecule, and increasing Bcl-2, an anti-apoptotic signaling molecule.^12^^,^^29^

Similarly, Cao et al.^30^ conducted western blot analyses and immunofluorescence of caspase-3, BAX, and Bcl-2 and reported that liraglutide exerted effects on the aforementioned proteins that favored cell viability in murine models.^30^ In addition, markers of neuroinflammation decreased in the liraglutide group, compared to the control, inhibiting inflammation and protecting dopaminergic neurons through the AMPK/NF-κB signaling pathway.^30^ Immunofluorescence testing revealed that levels of TH-positive neurons gradually increased with liraglutide concentration at significant values (p = .111 for 25 nmol/kg, p = .011 for 50 nmol/kg, and p = .001 for 100 nmol/kg).^30^

The foregoing findings are in accordance with Bu et al.^31^ and Liu et al.,^32^ who reported an attenuation of progressive neuronal loss with Ex-4.^31^^,^^32^ It has been suggested that autophagic pathways such as the phosphatidylinositol 3-kinase (PI3K)/protein kinase B (Akt) pathway may be involved in Ex-4-mediated degradation of pathological α-synucleinopathy.^31^ The upregulation of the pathway by PD was reversed by Ex-4, increasing markers that indicate active autophagy and decreasing markers that block autophagy.^31^

Alternatively, it has been proposed that decreased spontaneous dopaminergic neuron firing in PD is associated with the reduced expression of TH and a threat to neuronal survival.^32^ Liu et al.^32^ reported that administering Ex-4 increased the firing rate of 7 out of 14 nigral dopaminergic neurons from 2.20 ± 0.31 to 3.54 ± 0.41 Hz, mediated by ion channels.^32^ The activation of GLP-1 receptor-mediated neuronal firing within the SNpc may be associated with observable functions (e.g., motor activity) that alleviate some clinical features of PD.^32^

A pathophysiologic feature of PD includes microglia activation, which exacerbates dopaminergic neurodegeneration, oxidative stress, and neuroinflammation.^13^^,^^15^^,^^23^ Kim et al.^27^ and Elbassuoni and Ahmed^15^ assessed matrix metalloproteinase-3 (MMP-3) and malondialdehyde (MDA) levels, respectively, as biomarkers to evaluate microglial activation by neurodegeneration and oxidative stress at the cellular level.^15^^,^^27^ Kim et al.^27^ reported that Ex-4 prevented MPTP-induced loss of TH-positive SNpc neurons by 83% by attenuating the upregulation of MMP-3.^27^ MDA levels evaluated by Elbassuoni and Ahmed^15^ also significantly decreased with exenatide in a dose-dependent manner.^15^ In both studies, pro-inflammatory cytokines and striatal inflammatory biomarkers (e.g., TNF-α and IL-1β) were reduced with GLP-1RAs.^15^^,^^27^ It has also been reported that microglial-activated conversion of astrocytes into neurotoxic A1 phenotypes caused the loss of TH and Nissl neurons in the SNpc, which the GLP-1RA, NLY01, adequately prevented.^13^

### Clinical evidence of GLP-1RAs in PD

The search generated a total of three clinical studies evaluating the effect of GLP-1 RAs on either the prevention, treatment, or illness progression intervention of PD. A randomized, placebo-controlled study by Athauda et al.^33^ assessed the efficacy of exenatide compared to placebo in persons with PD.^33^ The primary outcome was a change in the Unified Parkinson’s Disease Rating Scale (MDS-UPDRS) part 3 subscale scores.^33^ Participants (*n* = 62) with moderate PD were randomized to either exenatide (2 mg subcutaneously once weekly) or placebo. At 48 weeks (the post-exposure period), MDS-UPDRS scores in the placebo group deteriorated by 1.7 points, while those in the exenatide group improved by 2.3 points.^33^ At 60 weeks, off-medication scores worsened by 2.1 points in the placebo group and improved by 1.0 points in the exenatide group (p = .0318).^33^ However, no significant differences were reported between the experimental and control groups on the other subsections of the MDS-UPDRS or in qualitative assessments (e.g., quality of life).^33^ No malaise-like symptoms attributable to changes in motor scores were noted.^33^

A separate phase 2, double-blind, randomized, placebo-controlled study compared lixisenatide to placebo as a disease-modifying intervention in PD by comparing MDS-UPDRS part 3 subscale scores in persons with PD.^34^ After 12 months, the mean score in the lixisenatide on-medication group exhibited a motor improvement of −0.04 points (a 14.9% improvement), while the placebo exhibited a worsening on the MDS-UPDRS by 3.04 points (an 18.8% worsening) (p = .007).^34^ Off-medication scores worsened in both groups, but the placebo group was inferior.^34^

A population-based cohort study utilized medical records to assess the incidence of PD among persons with T2DM prescribed GLP-1RAs with an overarching aim to determine whether GLP-1RAs mitigate the incidence of PD.^35^^,^^40^ The primary outcome was the first recorded diagnosis of PD, comparing patients who were prescribed metformin, sulfonylureas, or other oral antidiabetic medications.^35^ The crude relative risks for the incidence of PD were 0.83 (0.64–1.07), 0.54 (0.41–0.73), and 0.40 (0.24–0.66) for users of glitazone (GTZ), dipeptidyl peptidase-4 inhibitors (DPP4), and GLP-1 mimetics, respectively.^35^ The results indicated that an inverse relationship between the use of DPP4 inhibitors and GLP-1RAs and the onset of PD exists, with an approximate 36–60% relative decrease in the rate of PD onset compared to the use of other oral antidiabetic medications.^35^ A separate population-based cohort study recorded the crude incidence of PD amongst older persons with T2DM prescribed with GLP-1RAs or DPP4 inhibitors.^36^ These researchers found that the crude incidence rate of PD was lower among GLP-1RA users than DPP4 inhibitor users (2.85 versus 3.92 patients per 1000 person-years).^36^ Moreover, the research demonstrated that GLP-1RAs were associated with a 23% lower risk of PD than DPP4i users (HR, 0.77; 95% CI, 0.63–0.95).^36^

## Discussion

Herein, via systematic review, we identified replicated evidence extracted from both preclinical and clinical studies suggesting beneficial effects of select GLP-1RAs on cellular and molecular mechanisms as well as clinical aspects of PD.^12^^–^^15^^,^^22^^,^^27^^,^^29^^,^^31^^–^^33^^,^^36^ It has also been noted that the putative benefit of GLP-1RAs in animal models and persons living with PD extends across both motor and non-motor impairments.^12^^–^^15^^,^^22^^,^^27^^,^^29^^,^^31^^,^^33^^,^^36^ The reported benefits of GLP-1RAs on clinical aspects of PD, as well as the molecular and cellular effects, are aligned with prevailing models of disease pathogenesis in PD.^12^^–^^15^^,^^22^^,^^27^^,^^29^^,^^31^^–^^33^^,^^36^

More specifically, preclinical studies that sought to evaluate the effects of GLP-1RAs in MPTP- or α-synuclein-mediated Parkinsonian models reported meaningful restoration of motor function and protection of dopaminergic neurons.^11^^–^^15^ In addition, biomarkers of neuroinflammation and oxidative stress (e.g., proinflammatory cytokines) were also affected with the administration of GLP-1RAs, along with a suggestion of reduced microglial activation.^13^^,^^15^^,^^22^ Although clinical data are preliminary, using change in motor scores as an ancillary measure of disease progression suggests small benefits with some GLP-1RAs in treated PD subjects.^33^^,^^36^ The potential for GLP-1RAs to affect illness progression in PD has also been investigated in an observational study, wherein a significant decrease in PD incidence was observed in persons with T2DM prescribed GLP-1RAs.^40^

The findings of protective effects of GLP-1RAs on brain regions hypothesized to subserve the phenotypic characteristics of PD are in accordance with other lines of research, also suggesting benefit in other brain-based disorders (e.g., depression and substance use disorder).^41^^,^^42^ It is noteworthy that GLP-1RAs are not known to be causally related to any adverse CNS effects and/or suicidality, despite reports of suicide in some persons taking these agents.^43^^–^^45^ Moreover, there are separate agents (e.g., antioxidants, kinase inhibitors) that have been promising interventions for PD as demonstrated by several clinical trials.^46^

There are several methodological aspects that affect the interpretation of the studies that we identified as part of this systematic review. For example, there are axiomatic limitations with respect to extrapolating findings from preclinical models of PD to persons living with PD. In addition, although preliminary epidemiologic and controlled trials suggest GLP-1RAs may have protective and treatment effects in persons with PD, there is a need for adequately controlled, extensive replication studies with both acute and long-term outcomes evaluated to determine whether the effects can be replicated with meaningful effect sizes. Whether GLP-1RAs offer additional therapeutic effects as adjunctive agents to dopamine replacement therapy or other FDA-approved interventions is a vista for future research. Also, existing studies have primarily evaluated the effect of GLP-1RAs on motor symptoms in PD, there remains an open question as to the effect of these agents on other aspects of PD that are frequently encountered clinically (e.g., cognitive impairment, depressive symptoms).^47^ A final limitation is that we delimited eligibility to those studies evaluating the effect of GLP-1RAs when using the MPTP and a-synuclein model of Parkinson’s disease. We recognize there are other models of Parkinson’s disease (e.g., rotenone, 6-hydroxydopamine); notwithstanding, the great majority of studies evaluating the putative protective effects of GLP-1RAs were confined to MPTP and a-synuclein models; consequently, for consistency reasons, we decided to only focus on these models evaluating GLP-1RA effects.

## Conclusion

Some GLP-1RAs are capable of crossing the blood–brain barrier (e.g., liraglutide) and target molecular and cellular processes relevant to the pathophysiology of PD.^48^^,^^49^ These penetrating agents are reported to be most suitable for repurposing as CNS targets.^48^^,^^49^ Although the initial rationale to conduct studies with GLP-1RAs in PD was provided by observations of clinical and pathophysiological overlap with T2DM, the available preclinical and clinical evidence suggests that the putative benefits of these agents in PD are not limited to persons with T2DM and/or metabolic-related disorders. Priority research vistas are to conduct large, adequately powered, controlled studies in persons living with PD, with preferably head-to-head comparison with FDA-approved treatments for PD. In addition to the effects on motor outcomes, there is an equal priority to ascertain if GLP-1RAs may benefit measures of cognitive functions, which has been suggested in other brain-based disorders.^49^ Future research vistas should endeavor to ascertain whether beneficial effects of dual or triple incretin agonists on the clinical features and/or neural biology of PD have relative advantages over one aspect observed heretofore with incretin mono agonists.
